# Functional and Radiological Results of Proximal Femoral Nail Antirotation (PFNA) Osteosynthesis in the Treatment of Unstable Pertrochanteric Fractures

**DOI:** 10.3390/jcm7040078

**Published:** 2018-04-12

**Authors:** Ahmad M. Radaideh, Hashem A. Qudah, Ziad A. Audat, Rami A. Jahmani, Ibraheem R. Yousef, Abed allah A. Saleh

**Affiliations:** 1 Department of Orthopedics, King Abdullah University Hospital, Jordan University of Science and Technology, Irbid 22110, Jordan; ziadaudat@hotmail.com (Z.A.A.); dr.jahmany@yahoo.com (R.A.J.); ibraheem-yousef@live.com (I.R.Y.); drabedallah2013jor@gmail.com (A.a.A.S.); 2 Department of Orthopedics, Jordan Hospital, Amman 11152, Jordan; hashemq@yahoo.com

**Keywords:** pertrochanteric fracture, PFNA, intramedullary fixation, Harris Hip Score

## Abstract

Pertrochanteric femur fractures are considered amongst the most commonly encountered fractures in the geriatric age group. We evaluated radiographic and functional outcomes of patients with unstable pertrochanteric fractures treated with the proximal femur nail antirotation (PFNA). Between March 2013 and December 2015, fifty patients (28 male and 22 females with a mean age of 72.8 years (range, 20–94)) with unstable pertrochanteric fractures (AO 31.A2 and 31.A3) were fixed with the PFNA at our institution, and they were retrospectively evaluated. Forty one patients were treated with short PFNA and nine with long PFNA. Operative time ranged between 30 and 150 (average 73.60) min, blood loss ranged between 50 and 250 (average 80) milliliter and hospital stay ranged between 3 and 18 (6.86) days. The mean follow-up period was 18 months (range, 11–31). At final follow-up, solid union of all fractures had been achieved without any implant-related complications, the mean Harris Hip Score (HHS) was 79.34 ± 9.10 points and the mean neck-shaft angle was 127.2° ± 5.07°. No significant differences were encountered between the functional and radiographic outcomes of the PFNA with regards to the AO fracture classification and the implant version. PFNA is a recommended option for the treatment of unstable pertrochanteric fractures owing to its easy insertion, reduced blood loss, stable fixation and satisfactory functional and radiological outcomes.

## 1. Introduction

Pertrochanteric femoral fractures are common fractures amongst geriatric age group. Incidence of such fractures increased recently due to an increase in life expectancy worldwide [1]. These fractures are usually caused by minor traumas. They are classified according to AO/OTA classification system into three groups: 31.A1 pertrochanteric simple, 31.A2 pertrochanteric multifragmentary and 31.A3 intertrochanteric. Early mobility is the primary goal of treatment, to prevent the dreaded complications associated with immobilization [2]. Complications associated with pertrochanteric fractures are primarily related to the implant used rather than the union process owing to the strong healing potential of cancellous bone in the intertrochanteric region [3].

The best treatment option for unstable trochanteric fractures is still a matter of debate [4]. Different implants have been in use to provide a stable fracture fixation. They can be widely classified into two groups: extramedullary and intramedullary devices [5,6]. Dynamic hip screws are the most commonly used extramedullary devices for the treatment of hip fractures. Some studies, however, have reported that the sliding hip screw is not ideal for unstable pertrochanteric fractures and these studies have supported various alternative methods of stabilization for these more complex fractures [5,7,8].

Cephalomedullary devices are the favored treatment option for unstable pertrochanteric fractures, especially in the absence of medial buttressing at the proximal femur. They depend on the relative stability concept and treatment is usually performed by minimal invasive procedures. While numerous fixation devices are available for the treatment of pertrochanteric fractures, none of them are totally free of complications [9].

In 2004, the proximal femoral nail antirotation (PFNA), developed by the AO/ASIF group, was introduced as a one of the modern generation intramedullary nails. The unique characteristic of this implant is the use of a single blade with a large surface area. The inserted blade achieves an excellent fit through bone compaction and requires less bone removal compared with a screw. This feature provides optimal anchoring and stability especially, when inserted into osteoporotic bone and has been biomechanically proven to retard rotation and varus collapse [5,10,11].

Our study aims to assess the final functional and radiological results of the PFNA osteosynthesis for the treatment of unstable pertrochanteric fractures.

## 2. Experimental Section

Our retrospective study was performed on fifty patients (28 males and 22 females) with unstable pertrochanteric fractures at our institution King Abdullah University Hospital (KAUH), who were treated with the PFNA between March 2013 and December 2015. Their age ranged from 20 to 94 years with a mean of 72.8 years. A low-energy trauma was the cause of the fracture in 46 (92%) patients, most often a simple fall. Fractures were classified according to AO/OTA classification system. Type A2 fractures were the most common in this study and were seen in 37 (74%) patients and type A3 fractures were seen in 13 (26%) patients. Short PFNA was used in 41 patients (37 with A2 and 4 with A3 fractures), while long PFNA was used in 9 patients. The inclusion criteria were as follows: freshly closed unstable pertrochanteric fracture (31.A2-A3), age ≥ 20 years and performing definitive fracture fixation with PFNA within three days after admission. Multiple trauma patients, patients with severe cognitive impairment, severe disability and neoplastic pathological fractures were excluded from the study.

Surgery was performed as soon as the patient`s general health conditions allowed, average time waiting for surgery was three days. General or spinal anesthesia was used. All of the fractures were treated on the fracture table in a supine position under fluoroscopy guidance and the fractures were closely reduced and nailing was performed in all of them according to standard protocol for the PFNA. The nail blade angle of the device used was 125° in 36 patients and 130° in 14 patients.

All patients received 3 doses of prophylactic antibiotic therapy, starting 2 h before the operation. In addition, low-molecular-weight heparin was administered once daily for 4 weeks. Clinical and radiographic assessment was performed on admission to the hospital, at 1 month, at 3-month intervals in the first year and yearly thereafter. Operation time, intraoperative blood loss, the duration of hospitalization, postoperative blood transfusion and the surgical complications were recorded.

At final follow-up, functional level was estimated using the Harris Hip Score: scores < 70 Poor, between 70–89 Fair/Good, and above 90 considered as Excellent [12]. Radiological outcomes were assessed by measuring the neck-shaft angle of the proximal femur. Malunion was defined as a neck-shaft angle < 120°. Bone union is defined on radiographs as presence of callus, continuity of cortex and disappearance of fracture line. Non-union is defined as lack of bony consolidation after 12 months interval [13].

Rehabilitation protocol was started as soon as possible after surgery and weight bearing as tolerated was allowed immediately postoperatively using walking aids.

Statistical analysis was performed using SPSS (SPSS statistical package; version 23.0.0). Case characteristics were summarized using descriptive statistics, including the mean (SD), or median (minimum-maximum) for continuous variables. Independent samples t-test was used to measure the differences in bi-variate analyses. For all comparisons, statistical significance was assigned at *p* < 0.05.

## 3. Results

The mean (SD) follow up for the fifty patients enrolled in the study was 18.1 (4.9) months (Range, 11–31). The mean (SD) hospital stay was 6.86 (3.39) days (Range, 3–18). The mean intraoperative blood loss was 80 (54.92) ml (Range, 50–250). The mean operative time was 73.60 (31.28) min (range, 30–150) (Table 1). The mean operative time for patients who had A2 fracture was 62.1 min while that for patients with A3 fracture was 105 min (*p* value = 0.03) which was statistically significant owing to the frequent use of long PFNA in A3 fractures that require more surgical steps including the reaming of the medullary canal and the free hand technique for the distal locking screws. Postoperative transfusion of two units of packed RBCs was required in only eight patients.

A neck-shaft angle of more than 120 degrees was achieved in all patients immediately postoperatively.

At final follow-up, solid union of all fractures had been achieved. The mean neck-shaft angle for A2 fracture was 127.59° (5.14), while that for A3 fracture was 126.08° (4.91), with no significant difference (*p* value = 0.876) between the two fracture patterns. Moreover, the mean neck-shaft angle for long PFNA was 126.22° (5.85) while that for short PFNA was 127.41° (4.94) with no significant difference (*p* value = 0.561) between the two implant version.

The mean Harris Hip Score (HHS) for A2 fracture was 77.92 (9.27) points while that for A3 fracture was 83.38 (7.48) points with no significant difference (*p* value = 0.296) between the two fracture patterns. The mean HHS for short PFNA was 78.24 (8.86) points while that for long PFNA was 84.33 (8.96) points with no significant difference (*p* = 0.942) between the two implant versions (Table 2). No cases of implant failure had been recorded in our study (the incidence of blade cut out, blade cut through and nail breakage was 0%).

**Figure 1:**
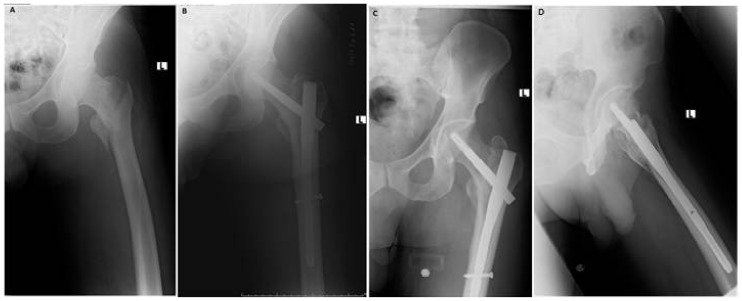
Type A2 fracture (**A**). Immediate postoperative AP radiograph showing a neck-shaft angle of 130° (**B**). The 6 months follow up AP radiograph showing a neck-shaft angle of 130° and union of the fracture (**C**). Lateral view at 6 months follow up (**D**).

**Figure 2:**
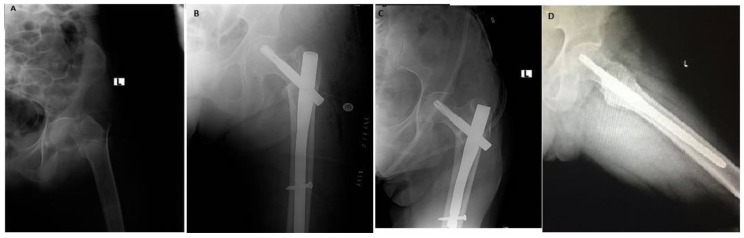
Type A3 fracture (**A**). Immediate postoperative AP radiograph showing a neck-shaft angle of 120° (**B**). 4 months follow up x-ray showing varus collapse with neck shaft angle of 115° (**C**). Lateral view at 4 months follow up (**D**).

At final follow-up, neck shaft angle was maintained above 120° in 44 (88%) patients and only six (12%) patients developed secondary varus collapse (neck-shaft angle < 120 degree) (Figure 1 and Figure 2). The average HHS was 79.3 points. Harris Hip Scores was excellent in 9 (18%) patients, good/fair in 32 (64%) patients and poor in 9 patients (18%).

## 4. Discussion

Stable fixation for pertrochanteric fractures is the primary goal of treatment. It allows early mobilization and restoration of limb function. Early operation is crucial for improving functional outcomes and avoiding serious complications associated with patients` immobilization. The PFNA device is a reliable internal fixator, it can share a large axial load, its helical blade achieves an excellent fit through bone compaction with less bone removal. The inserted blade prevents rotation by locking with the nail and accordingly, it may be a more suitable implant for unstable trochanteric fractures especially in the presence of osteoporosis. Biomechanical studies have shown that the blade has a higher resistance to head collapse than commonly used screw design [11,14]. The first biomechanical study of the PFNA device suggests that the inferior position of the helical blade in the frontal plane and center position in the sagittal plane is superior to the center-center position in both planes and provides better biomechanical stability [15].

In our study, the mean operative time for patients with A2 fracture was 62.1 min while for A3 fracture it was 105 min (*p* value = 0.03), which was statistically significant owing to the frequent use of long PFNA in A3 fracture which requires more surgical steps including the reaming of the medullary canal and the free hand technique for the distal locking screws. In comparison with other studies, the mean surgery time was 68 min [16], 67 min [17] and 20 min [18].

According to literature, the perioperative and postoperative technical complications described in the trochanteric fractures treated with the gamma nail were solved with the development of the PFNA [19,20,21]. In a study by Aguado-Maestro et al., there were 200 patients of pertrochanteric fractures treated with PFNA, they found that helical blade device reduced the rate of cut through and cut out in pertrochanteric fractures and accurate placement of the helical blade was a key factor to prevent mechanical failures and they reported the incidence of cut out was 1% [22]. While the cut-out rates were found to be 2% in a study by Takigami et al., 4.7% in a study by Şahin et al. and 7.9% in a study by Zhang et al. [13,18,23]. The rates of femoral head perforation were found to be 1.4% in a study by Karapınar et al. and 1.2% in a study by Simmermacher et al. [24,25]. In consistence with previous reports, in this study, the incidence of blade cut-out and femoral head perforation was 0%.

In a series of 225 cases with intertrochanteric fractures fixed with a PFNA, investigators described that varus collapse occurred in 4.9% of cases [26]. While the varus collapse rate was found to be 5.8% in a study by Zhang et al. [13]. In this study, 88% of patients had neck-shaft angle maintained above 120 degrees, while varus collapse occurred in 12% of cases. When interpreting relatively high failure rates of internal fixation (collapse of femoral head) in the current study, several factors have been identified. Firstly, the failure is due to inadequate reduction and/or poorly placed helical blade of the PFNA (in superior-center position on radiographs). Additionally, early functional exercise, high activity levels, increasing age and presence of osteoporosis might increase the risk of implant failure. 

Regarding clinical outcomes in this study, the mean HHS at final follow-up was 79.3 ± 9.10 points. Zhang et al. reported 139 PFNA-treated cases and found the mean HHS was 72.4 ± 7.20 [13]. The mean HHS was found to be 82 points in a study by Macheras et al. [27]. In our series, 41 (82%) patients had excellent to fair HHS and only nine (18%) patients had poor HHS. The functional results were acceptable and comparable with the results of retrospective studies by Simmermacher et al. [25] and Mereddy et al. [11], who also observed restoration of preoperative mobility in approximately 56% to 80% of patients treated with the PFNA.

In our study, there were limitations inherent in the methodology used because it was a retrospective study with no control group.

## 5. Conclusions

The PFNA is a superior implant for the treatment of unstable pertrochanteric fractures owing to its easy insertion, reduced blood loss, less complications, stable fixation and satisfactory functional and radiological outcomes.

No significant differences were encountered between the clinical and radiographic outcomes of the PFNA with regards to the AO fracture classification and the implant version.

## Figures and Tables

**Table 1:** The demographic and perioperative characteristics of patients with unstable pertrochanteric fractures who were treated with the PFNA.

Variable	Value
Patients No.	50
Age: mean (range)	72.8 (20–94)
Gender: male/female	28/22
Type of fracture	
A2	37
A3	13
Mechanism of injury	
Simple fall at home	46
Traffic Accident	4
Mean blood loss (mL) ± SD	80 ± 54.92
Mean operative time (min) ± SD	73.60 ± 31.28
Mean hospital Stay (days) ± SD	6.86 ± 3.39

**Table 2:** Comparisons of final functional and radiological outcomes amongst patients with unstable pertrochanteric fractures who were treated with the PFNA.

	Number	Mean Harris Hip Score ± SD	Mean Neck-Shaft Angle ± SD
Fracture type			
31A2	37	77.92 ± 9.27	127.59 ± 5.14
31A3	13	83.38 ± 7.48 *p*-value 0.27	126.08 ± 4.91 *p*-value 0.88
Fixation device			
Long PFN	9	84.33 ± 8.96	126.22 ± 5.85
Short PFN	41	78.24 ± 8.86 *p*-value 0.94	127.41 ± 4.94 *p*-value 0.56
